# Shuangshen Granule Regulates Tumor Cell Exosomes Through MIF‐miR‐34a‐KLF4 Pathway and Affects Macrophage Polarization Against Lung Cancer

**DOI:** 10.1155/humu/5626229

**Published:** 2026-04-15

**Authors:** Runzhi Qi, Yi Li, Zhongning He, Yuwei Zhao, Qi Huang, Yuxuan Liu, Yongxia Hou, Zhan Shi, Baojin Hua, Bo Pang, Yefan Zhang, Qiujun Guo

**Affiliations:** ^1^ Department of Oncology, Guang′anmen Hospital, China Academy of Chinese Medical Sciences, Beijing, China, cacms.ac.cn; ^2^ International Medical Department, Guang′anmen Hospital, China Academy of Chinese Medical Sciences, Beijing, China, cacms.ac.cn; ^3^ Department of Otolaryngology, Guang′anmen Hospital, China Academy of Chinese Medical Sciences, Beijing, China, cacms.ac.cn; ^4^ Institute of Basic Research in Clinical Medicine, China Academy of Chinese Medical Science, Beijing, China, cacms.ac.cn; ^5^ School of Traditional Chinese Medicine, Beijing University of Chinese Medicine, Beijing, China, bucm.edu.cn; ^6^ Department of Oncology, Jinan Laiwu People′s Hospital, Jinan, China; ^7^ Department of Oncology, Xiyuan Hospital, China Academy of Chinese Medical Sciences, Beijing, China, cacms.ac.cn; ^8^ Department of Liver and Gallbladder Surgery, National Cancer Center/National Clinical Research Center for Cancer/Cancer Hospital, Chinese Academy of Medical Sciences and Peking Union Medical College, Beijing, China, cacms.ac.cn

**Keywords:** exosomes, lung cancer, MIF, SSG, TAMs

## Abstract

The incidence and mortality of lung cancer are among the highest in the world, involving a variety of complex pathological mechanisms and processes. Previous studies have demonstrated that the tumor immunosuppressive microenvironment (TIME) plays a key role in tumor progression, promoting tumor invasion and metastasis and leading to resistance to therapy. Tumor‐associated macrophages (TAMs) are important cell components in TIME, and their phenotypes affect the prognosis of tumors. Tumor‐derived exosomes carrying specific information as a medium of crosstalk between macrophages and tumor cells are considered to be an underexplored information transfer medium affecting tumor progression and tumor microenvironment remodeling. In the previous study, we found that Shuangshen granule (SSG) can reduce the formation of lung tumors in mouse models of spontaneous lung cancer, but the specific mechanism of SSG inhibiting lung cancer has not been solved. Starting from the exosomes of tumor cells, we established the M1‐like TAMs overexpression mouse lung cancer transplantation tumor model and constructed MIF^+/+^ Lewis stable transfection cell line and shMIF Lewis stable transfection cell line to analyze the specific molecular biological mechanism of SSG inhibiting lung cancer. The experimental results showed that SSG significantly reduced the volume and mass of Lewis lung cancer xenografts in mice, and its inhibitory effect on Lewis lung cancer was related to the increase in the proportion of M1‐like TAMs. Flow cytometry and western blot revealed that SSG can increase the proportion of CD8^+^ central memory T cells, reduce the proportion of CD8^+^ effector memory T cells, reduce the proportion of CD4^+^CD25^+^Foxp3^+^ Treg cells, and play a role in regulating the tumor microenvironment. In vitro experiments further showed that SSG drug–containing serum could inhibit the expression of MIF protein and RNA in MIF^+/+^ Lewis cells and increase the expression of miR‐34a in MIF^+/+^ Lewis cell exosomes. Interestingly, we also found that MIF^+/+^ Lewis cell–derived exosomes after SSG‐containing serum intervention can reverse the increase in the proportion of M2‐like TAMs and increase the proportion of M1‐like TAMs. Subsequent gene modification experiments showed that MIF^+/+^ Lewis cell exosomes can increase the expression of the KLF4 gene in macrophages in vitro. In conclusion, our study revealed that SSG can regulate the polarization of TAMs through lung cancer cell–derived exosomes, and its mechanism is related to the MIF‐miR‐34a‐KLF4 pathway.

**Summary:**

• SSG can inhibit the proliferation of lung cancer, which is related to the antitumor mechanism involving macrophages.
• The poor prognosis of lung cancer caused by macrophage migration inhibitory factor (MIF) is related to the TAM phenotype, and SSG can inhibit lung cancer by inhibiting MIF.
• SSG regulates the tumor cell MIF‐MiR‐34a exosome pathway and regulates the polarization and function of TAMs.

## 1. Introduction

Lung cancer is the main cause of cancer‐related deaths in the world. The latest research data show that about 1.8 million people die of lung cancer every year, accounting for 18.7 of the total number of cancer deaths, which is a great threat to human health [1]. In fact, while lung cancer seriously affects public health, it also brings huge “financial toxicity,” resulting in a huge increase in global medical expenses [2]. NSCLC is the major type of lung cancer, which accounts for 85%–90% of lung cancer patients. Though targeted therapies and immunotherapies have made great strides in recent years, the prognosis of NSCLC is still poor, with a dismal 5‐year survival rate of < 15% [3]. Therefore, it is important to explore novel ways to improve clinical outcomes.

TIME plays a vital role in tumor progression, by not only promoting invasion and metastasis but also leading to resistance to tumor treatments. The composition of TIME is very complex, with large differences in immune cell composition, functional status, and spatial distribution heterogeneity [4]. Among TIME, TAMs represent up to 50% of the tumor mass, and most TAMs have the M2 phenotype due to the signals in the TIME. TAMs lead to a poor clinical prognosis, promote TIME progression of lung cancer via recruiting regulatory T cells (Tregs), inducing immune suppressive factors (IL‐10, TGF‐*β*, MMP‐9, Arg1, etc.) [5]. Although research on immune checkpoint inhibitors is hot these days, immunotherapy against TAMs remains to be further studied. Besides the cytokines and signal pathways, exosomes are a novel cell‐to‐cell signaling method with an average size of 100 nm structure that can transport proteins, lipids, and nucleic acids and can also lead to tumor progression and TIME regulation [6]. Tumor‐derived exosomes can affect the phenotype and function of macrophages, and miRNAs contained in exosomes induce tumor‐associated macrophages by regulating signaling pathways during tumor progression [7]. Therefore, modulation of tumor cell exosomes to intervene in TAM polarization is regarded as a promising tumor treatment strategy.

Although many studies have shown that M1‐like TAMs have antitumor effects, during tumor progression, M1‐like TAMs will gradually polarize into tumor‐promoting M2 [8]. Therefore, targeted regulation of TAMs has become a new strategy for cancer therapy. At present, the research on the regulation of TAM function by multiple signaling pathways has entered the clinical research stage, but in fact, the development of targeted therapeutic drugs for this pathway still has a long way to go [9]. As an important means of tumor treatment, Chinese medicine is considered to be a very important tumor treatment strategy in reducing the clinical symptoms caused by tumors and prolonging the survival of patients with postoperative and advanced cancer. We formerly reported Feiliuping ointment, which has been used in clinical practice for nearly 30 years in patients with NSCLC, on improving the survival time of NSCLC patients, inhibiting tumor cell epithelial–mesenchymal transition, and regulating the PI3K/AKT/NF‐*κ*B pathway in TIME and immune cells [10]. SSG is composed of traditional Chinese herbs, such as *Cordyceps sinensis*, American ginseng, and *Panax notoginseng*, and is a major component of Feiliuping ointment [11]. SSG has been proven to reduce tumor weight and lung metastasis by weakening the differentiation of bone marrow cells (BMCs) into myeloid‐derived suppressor cells (MDSCs) [12]. However, the mechanisms of SSG in regulating exosomes and TAMs in TIME to inhibit lung tumor progression are yet unclear.

In this study, we found that SSG can regulate the tumor cell exosomes by regulating the MIF‐miR‐34a‐KLF4 pathway, then regulate the polarization of macrophages, and ultimately inhibit lung cancer cells. In vivo studies have also shown that SSG can inhibit the growth of Lewis lung cancer xenografts, and more importantly, SSG has been shown to be involved in regulating the tumor microenvironment and regulating the polarization of mouse TAMs to M1 type. Our study provides a scientific basis for the prevention and treatment of lung cancer from the perspective of molecular biology and also highlights the key role of MIF‐miR‐34a‐KLF4 as a potential therapeutic target in the prevention of lung cancer metastasis.

## 2. Materials and Methods

### 2.1. Drug Preparation and Quality Control

SSG was extracted from a mixture of three herbs, including *Panax notoginseng*, *Panax quinquefolius*, and *Cordyceps sinensis*, by the refluxing extraction method. The chemical constituents of SSG were analyzed using an ultrahigh‐performance liquid chromatography (UHPLC) system coupled with Orbitrap HRMS (Thermo Fisher Scientific, United States).

Briefly, 1.0 g Shuangshen granule was accurately weighed and dissolved in methanol to 25 mL. The redissolved methanolic solution was filtered with a 0.22‐*μ*m filter before being injected into the UHPLC‐Q‐Exactive Orbitrap HRMS system. The chromatographic analysis was performed with an Acquity UPLC BEH C18 column (2.1 × 100 mm, 1.7 *μ*m, Waters Corporation, United States) using a Dionex Ultimate 3000 UHPLC system (Thermo Fisher Scientific, United States). Methanol (B) and 0.1% formic acid aqueous solution (A) were used as the mobile phase. The flow rate was 0.30 mL/min at 40°C applied with a linear gradient as follows: 0.0–2.0 min, 4%B; 2.0–6.0 min, 4%–12%B; 6.0–38.0 min, 12%–70%B; 38.0–38.5 min, 70%B; 38.5–39.0 min, 70%–95%B; 39.0–43.0 min, 95%B; and 43.0–45.0 min, 4%B. An aliquot volume (5 *μ*L) of the filtered samples was injected for analysis. The MS analysis was performed using both positive and negative modes equipped with a heated electrospray ionization (HESI) source. MS parameters were set as follows: spray voltage of 3.5 kV in positive mode and 2.5 kV in negative mode, heat temperature of 300°C, sheath gas flow rate of 35 arbitrary units, auxiliary gas of 13 arbitrary units, and capillary temperature of 320°C. The full scan data were recorded at a mass resolving power of 70,000 FWHM, while data‐dependent MS2 spectra were acquired at 17,500 FWHM.

### 2.2. Animal Experiments

Four‐week‐old male SPF C57BL/6 mice were obtained from the Beijing Vital River Laboratory Animal Technology Co., Ltd. The mice were raised in the Laboratory of Oncology, Guang′anmen Hospital, China Academy of Chinese Medical Sciences, under specific pathogen‐free conditions with the ambient temperature of 20°C–24°C and 45%–55% relative humidity and given sterilized food and water. The rearing facility was maintained on a 12‐h light–dark cycle. To establish lung cancer in mice, a fully mixed Lewis cell suspension was injected subcutaneously in the right axilla of mice at a density of 2 × 10^6^. The mice were randomized into five groups (*n* = 50), including high‐dose SSG (2.08 g/kg/day), medium‐dose SSG (1.04 g/kg/day), low‐dose SSG (0.52 g/kg/day), purified water (gavage), and cyclophosphamide (4 mg/mL, intraperitoneal injection on Days 5, 9, 13, and 17). Throughout the treatment, mice were weighed, and their tumors were measured with a caliper every 3 days. Tumor volumes (*V*) were calculated using the following formula: *V* = (length) × (width)^2^/2. Three weeks later, 0.4% sodium pentobarbital 30–50 mg/kg was intraperitoneally injected to anesthetize mice, blood was collected from the submandibular venous plexus, and after 4 h of rest, the serum was obtained by centrifugation. After blood collection, the mice were killed by dislocation of the cervical vertebrae, and the tumor tissue and spleen were collected. Primary cells were isolated from fresh tumors and spleens and subjected to TAM polarization analysis as indicated above by flow cytometry and immunofluorescence.

Four‐week‐old male SPF C57BL/6 mice were obtained from the Beijing Vital River Laboratory Animal Technology Co., Ltd. All in vivo experiments were also reviewed and approved by the company. The mice were raised in the tumor laboratory of Guang′anmen Hospital, China Academy of Chinese Medical Sciences, under sterile conditions, and the parameters of the feeding environment were as described above. To establish the Lewis and M1 cell mixed tumor in mice, a fully mixed Lewis and M1 cell suspension was injected subcutaneously in the right axilla of mice at a density of 2 × 10^6^. The mice were randomized into four groups (*n* = 40), including Lewis (purified water gavage), Lewis+SSG (2.08 g/kg/day by gavage), Lewis+M1 (coinjection of Lewis cells with M1 phenotype RAW264.7 at the ratio of 4:1), and Lewis+M1+SSG (Lewis cells and M1 phenotype RAW264.7 coinjection, followed by SSG treatment). Throughout the treatment, mice were weighed, and their tumors were measured with a caliper every 3 days. Tumor volumes (*V*) were calculated using the following formula: *V* = (length) × (width)^2^/2. Three weeks later, 0.4% sodium pentobarbital 30–50 mg/kg was intraperitoneally injected to anesthetize mice, blood was collected from the submandibular venous plexus, and after 4 h of rest, the serum was obtained by centrifugation. After blood collection, the mice were killed by dislocation of the cervical vertebrae, and the tumor tissue and spleen were collected. Primary cells were isolated from fresh tumors and spleens and subjected to TAM polarization analysis as indicated above by flow cytometry and immunohistochemical staining.

In order to establish the transplantation tumor model of mice in different groups, MIF^+/+^ Lewis, shMIF Lewis, and Lewis cells were taken, and the cell suspension was prepared and injected into the right armpit of mice at a density of 2 × 10^6^. Mice were randomly divided into five groups (*n* = 50), including Lewis (purified water gavage), Lewis+SSG (2.08 g/kg/day by gavage), MIF^+/+^ Lewis (purified water gavage), MIF^+/+^ Lewis+SSG (2.08 g/kg/day by gavage), and shMIF Lewis (purified water gavage). Throughout the treatment, mice were weighed, and their tumors were measured with a caliper every 3 days. Tumor volumes (*V*) were calculated using the following formula: *V* = (length) × (width)^2^/2. Three weeks later, 0.4% sodium pentobarbital 30–50 mg/kg was intraperitoneally injected to anesthetize mice, blood was collected from the submandibular venous plexus, and after 4 h of rest, the serum was obtained by centrifugation. After blood collection, the mice were killed by dislocation of the cervical vertebrae, and the tumor tissue and spleen were collected. Primary cells were isolated from fresh tumors and analyzed for TAM polarization by flow cytometry as described above.

This study complies with the ARRIVE Guidelines for animal research and is conducted strictly in accordance with the recommendations of the Management and Use Guidelines for Laboratory Animals of the Guang′anmen Hospital of the China Academy of Chinese Medical Sciences. Animal procedures are carried out according to the approved plan of the Animal Ethics Committee of the Guang′anmen Hospital of the China Academy of Chinese Medical Sciences (No. IACUC‐GAMH‐2025‐033).

### 2.3. Cell Culture and Induction

The mouse lung cancer cell line Lewis and the mouse macrophage cell line Raw264.7 were obtained from the Chinese National Infrastructure of Cell Line Resource (Beijing, China). Lewis and Raw264.7 were cultured in DMEM/high, respectively, supplemented with 10% fetal bovine serum, 100 U/mL penicillin, and 100 mg/mL streptomycin. All the cells were maintained at 37°C in a humidified incubator containing 5% CO_2_. A concentration of 100 ng/mL lipopolysaccharide (LPS) (tlrl‐3pelps, InvivoGen, United States) and 100 ng/mL IFN‐*γ* (315‐05, PeproTech, United States) was used to induce the transformation of Raw264.7 into M1 phenotype. A concentration of 40 ng/mL IL‐4 (214‐14, PeproTech, United States) and 40 ng/mL IL‐13 (210‐13, PeproTech, United States) was used to induce Raw264.7 macrophages into M2‐like TAMs.

### 2.4. Flow Cytometry Assay

Cells were harvested, washed, and resuspended in 100 *μ*L 2% FBS·PBS solution at a density of 1 × 10^6^ cells. For the detection of M1 and M2 polarization, PE rat antimouse F4/80 clone T45‐2342 (565410, BD Pharmingen, United States), Alexa Fluor 647 rat antimouse CD206 clone MR5D3 (565250, BD Pharmingen, United States), and BB700 rat antimouse CD86 (742120, BD Pharmingen, United States) were used. For identification of Tregs and memory T cells, cells were incubated with PerCP rat antimouse CD8a (553036, BD Pharmingen, United States), PE rat antimouse CD44 clone IM7 (553134, BD Pharmingen, United States), APC rat antimouse CD62L clone MEL‐14 (553152, BD Pharmingen, United States), FITC rat antimouse CD4 (557307, BD Pharmingen, United States), APC rat antimouse CD25 (558643, BD Pharmingen, United States), and PE rat antimouse Foxp3 (560408, BD Pharmingen, United States) using flow cytometer. After incubation, cells were washed with 2% PBS and subjected to the BD Accuri C6 flow cytometer (BD Pharmingen).

### 2.5. Immunofluorescence

For immunofluorescence detection, paraffin sections were first dewaxed by placing the tumor tissue sections in Xylene I for 20 min, Xylene II for 20 min, and Xylene III for 20 min. Then, the sections were dehydrated with gradient concentrations of ethanol and immersed for 3 min each time, followed by washing with distilled water. The tumor tissue sections were placed in a pressure cooker containing EDTA antigen repair buffer for antigen repair, and the timer was started after the pressure cooker began venting for 3 min. After natural cooling, wash the slides with PBS. Add 1% BSA to cover the slides evenly, and incubate at room temperature for 60 min. Incubate the tissue sections with antimannose receptor (CD206) antibody (ab64693, Abcam, United States) and anti‐CD86 (GL‐1) antibody (ab119857, Abcam, United States) overnight at 4°C, followed by incubation with antirabbit IgG (H+L), F(ab′)2 fragment (Alexa Fluor 594 Conjugate) (8889, Cell Signaling Technology, United States), and antimouse IgG (H+L) (Alexa Fluor 488 conjugate) (4416, CST, United States) for 2 h. Subsequently, add DAPI (C1005, Beyotime, Shanghai, China) nuclear stain to the sections. Wash the sections, observe the fluorescence of the target antibody and the nucleus, seal with an antifluorescence quenching agent, observe the staining effect, and take photos using the laser scanning confocal microscope (FV 1000, Olympus, Japan).

### 2.6. Immunohistochemistry

Place the dewaxed sample in 3% hydrogen peroxide, incubate in the dark at room temperature for 10 min, and then remove and wash with PBS. Subsequently, an appropriate amount of 3% BSA blocking solution is added to the histochemical circle to cover the sample uniformly and incubated at room temperature in a wet box for 1 h, removing the excess blocking solution from the sample. The primary antibody is diluted according to the antibody instruction manual, and the diluted primary antibody is added to the sample and incubated overnight at 4°C.The sample is placed at room temperature for 1 h, and the primary antibody is recovered after rewarming. A secondary antibody corresponding to the species of the primary antibody is added to the sections, covered over the sample, and incubated at room temperature for 45 min. Next, through the use of diaminobenzidine (DAB) staining and gentle hematoxylin restaining, the reaction time is flexibly adjusted under microscopic observation. Finally, neutral resin is used to mount the slides, placed in a fume hood or on a paperboard to dry, and observed under a fluorescence microscope.

### 2.7. Western Blotting

Pretreat the cells to be detected according to the experimental requirements, and then, lyse them with RIPA (C1053, Applygen, Beijing, China). Protein concentration was quantified with the BCA Protein Assay Kit (23227, Thermo Fisher, United States). Load an equal amount of 40 *μ*g protein samples onto an SDS‐PAGE gel for electrophoretic separation, and then, transfer the separated proteins to a polyvinylidene fluoride microporous membrane (P2120, Applygen, Beijing, China). Specific antibodies are used to detect the target signal, and then, the signal is amplified and enhanced with corresponding secondary antibodies. The primary antibodies included iNOS (ab115819, Abcam, United States), ARG1 (93668S, CST, United States), TGF‐*β* (ab215715, Abcam, United States), VEGF (66828‐1‐Ig, Proteintech, United States), MIF (ab187064, Abcam, United States), MMP‐9 (ab228402, Abcam, United States), GAPDH (#5174, Cell Signaling Technology, United States), CD63 (ab216130, Abcam, United States), TSG101 (102286‐T38, SinoBiological, Beijing, China), and CD9 (ab92726, Abcam, United States). Finally, the bands were imaged through the luminata crescendo (WBKLS0100, Millipore, United States).

### 2.8. Cell Transfection

Our previous in vivo study found that MIF was closely associated with polarization of TAMs. Therefore, in this study, we mainly explored its roles in vitro. We constructed Lewis cells overexpressing MIF and Lewis cells with low expression of MIF. Briefly, the pHS‐AVC‐LY018 and pHS‐ASR‐LY058, as well as the corresponding blank control vectors pHS‐BVC‐LW345 and pHS‐ASR‐LW429, were prepared. The Lewis cells were seeded into a 24‐well plate at a density of 3 × 10^4^ cells/well and cultured overnight. The next day, the medium was changed to Dulbecco′s Modified Eagle Medium (11965118, Gibco, United States), and the cells were transfected with 10 *μ*g vectors. After being transfected overnight, the medium was replaced with complete medium and cultured for another 48 h. The level of MIF in the cells with different treatments was measured by RT‐qPCR to evaluate the cell transfection efficiency. The sequences of MIF and shMIF are shown in Table 1.

**Table 1:** MIF and shMIF sequences.

Target gene	Target sequence
MIF	5 ^′^‐ATGCCTATGTTCATCGTGAACACCAATGTTCCCCGCGCCTCCGTGCCAGAGGGGTTTCTGTCGGAGCTCACCCAGCAGCTGGCGCAGGCCACCGGCAAGCCCGCACAGTACATCCAGTGCACGTGGTCCCGGACCAGCTCATGACTTTTAGCGGCACGAACGATCCCTGCGCCCTCTGCAGCCTGCACAGCATCGCAAGATCGGTGGTGCCCAGAACCGCAACTACAGTAAGCTGCTGTGTGGCCTGCTGTCCGATCGCCTGCACATCAGCCCGGACCGGGTCTACATCAACTATTACGACATGAACGCTGCCAACGTGGGCTGGAACGGTTCCACCTTCGCT‐3 ^′^
shMIF	5 ^′^‐GCTCCACGTAGTGTTCTGTGT‐3 ^′^

### 2.9. RT‐qPCR Analysis

Total RNA was extracted using RNeasy RNA Kit (#74104, Qiagen, Germany). The RNA quantification was performed using a Nanodrop 2000/2000C Spectrophotometer (Thermo Fisher, United States). Reverse transcription was performed using the RevertAid M‐MuLV RT (EP0441, Thermo Fisher, United States). DNA was quantified using Power Up SYBR Green Master Mix (A25742, Applied Biosystems, United States). The specific sequences of the primers used are shown in Table 2. Quantitative normalization of target cDNA was performed for each sample using *β*‐actin, U6, and GAPDH as internal controls. The relative levels of MIF versus *β*‐actin, MIF‐shRNA versus *β*‐actin, miR‐34a versus U6, and Krüppel‐Like Factor 4 (KLF4) versus GAPDH were determined by the comparative CT (2^−*Δ* *Δ*CT^) method.

**Table 2:** The specific sequences of the primers.

Target gene	Target sequence
MIF	5 ^′^‐TCCGCCACCATGCCTATGTTCA‐3 ^′^ (forward) and 5 ^′^‐ACCACCGATCTTGCCGATGCT‐3 ^′^ (reverse)
MIF‐shRNA	5 ^′^‐CGGGTTTATTACAGGGACAGCAG‐3 ^′^ (forward) and 5 ^′^‐AAGAACGTTCACGGCGACTA‐3 ^′^ (reverse)
miR‐34a	5 ^′^‐TGGCAGTGTCTTAGCTGGTTG‐3 ^′^ (forward) and 5 ^′^‐GTCGTATCCAGTGCAGGGTCCGAGGTATTCGCACTGGATACGACACAACCAG‐3 ^′^ (reverse)
KLF4	5 ^′^‐CTGAACAGCAGGGACTGT‐3 ^′^ (forward) and 5 ^′^‐GTGTGGGTGGCTGTTCTTTT‐3 ^′^ (reverse)
*β*‐actin	5 ^′^‐GGAGGGGGTTGAGGTGTT‐3 ^′^ (forward) and 5 ^′^‐GTGTGCACTTTTATTGGTCTCAA‐3 ^′^ (reverse)
U6	5 ^′^‐CTCGCTTCGGCAGCACA‐3 ^′^ (forward) and 5 ^′^‐AACGCTTCACGAATTTGCGT‐3 ^′^ (reverse)
GAPDH	5 ^′^‐GTGAAGGTCGGTGTGAACGGATT‐3 ^′^ (forward) and 5 ^′^‐CGTGAGTGGAGTCATACTGGAACAT‐3 ^′^ (reverse)

### 2.10. Exosome Isolation and Characterization

After collecting the cell culture supernatant, first perform a 30‐min differential centrifugation at 2000 g. Subsequently, the supernatant was purified by filtration using a 0.22‐*μ*m filter unit (Millipore, United States) and then placed in a 4°C environment for 2 h of ultracentrifugation at 120,000 g. Discard the supernatant, resuspend the precipitated particles in precooled PBS buffer, and then recentrifuge for 2 h at 4°C and 120,000 g. The exosome particles obtained are resuspended in PBS and stored −80°C for future use. The concentration of exosomes was determined by the BCA protein quantification method, and their morphological characteristics were observed and verified by transmission electron microscopy. The specificity of exosomes is confirmed by detecting the expression of three marker proteins: CD63, TSG101, and CD9. The nanoparticle tracking analyzer equipped with a 488 nm laser (ZetaView PMX 110, Particle Metrix, Germany) was used to detect the sample. The concentration of the outer shell and the hydrodynamic diameter were calculated based on the particle motion trajectory.

The exosome used for cocultivating to induce M1 and M2 phenotype TAMs was extracted using the exoEasy Maxi Kit (Qiagen, Germany) according to the manufacturer′s instructions. Briefly, the culture supernatants were collected and filtered. Then, buffer XBP was added to the sample at a ratio of 1:1, and the tube was gently inverted five times to mix well. The mixture was added to the exoEasy spin column and centrifuged at 500 g for 1 min. After discarding the flow‐through, 10 mL buffer XWP was added to the collection tube, which was then centrifuged at 5000 g for 5 min to remove residual buffer from the column. Next, the flow‐through, together with the collection tube, was discarded, and a fresh collection tube was used to transfer the spin column. A 400 *μ*L buffer XE was added to the membrane and incubated for 1 min, after which the eluate was collected by centrifuging at 500 g for 5 min. The eluate was reapplied to the exoEasy spin column membrane and incubated for 1 min. Lastly, the eluate was centrifuged at 5000 g for 5 min and then transferred to another fresh tube.

### 2.11. Enzyme‐Linked Immunosorbent Assay (ELISA)

To investigate the effect of exosomes on the expression of IL‐10 and TNF‐*α* in cell supernatant, ELISA was performed using the QuikCyto Mouse TNF‐*α* ELISA kit (EMC102a, NeoBioscience Technology Co., Ltd., China) and the QuikCyto Mouse IL‐10 ELISA kit (EMC005, NeoBioscience Technology Co., Ltd., China). Absorbance was measured at 450 nm using a Microplate Reader (SynergyHT, BioTek, United States) within 3 min after completion of the reaction. Cytokine concentrations were scaled according to the standard curve.

### 2.12. Data Acquisition and Difference Analysis

As the largest cancer genetic information database currently available, the TCGA database (https://portal.gdc.cancer.gov/) stores abundant cancer‐related data, covering various types such as gene expression data, copy number variations, and single‐nucleotide polymorphisms (SNPs). We downloaded the original mRNA expression data and miRNA data of LUAD (lung adenocarcinoma) data and collected a total of 527 specimens. The normal group (*n* = 20) and the tumor group (*n* = 507) were used to analyze the sample data. For the miRNA detection data of 527 cases of LUDA, the R package “limma” was used to perform differential comparison analysis between cancer and normal and to obtain differentially expressed genes. The differential screening threshold was set as adj.*p*.val < 0.05 and |log fold change|(|log FC|) > 1. The differential miRNA of LUAD and the differential miRNA of mouse exosomes (|log2.Fold_change.| > 4) were screened, and the intersection was taken.

### 2.13. Immune Cell Infiltration Analysis

The tumor‐infiltrating immune cell analysis tool Tumor Immune Estimation Resource (TIER) was used to analyze the immune infiltration of LUAD samples. For the proportion of macrophage infiltration and the expression data of miR34a‐5p and regulatory target genes, the difference between macrophage infiltration groups and the correlation between expression and infiltration were calculated according to the high‐ and low‐expression groups of miR34a‐5p in the sample.

### 2.14. Statistical Analysis

Data from in vivo and in vitro experiments were presented as mean ± standard deviation (SD), and statistical analyses were performed using Statistical Product and Service Solutions (SPSS) 20.0 software (International Business Machines Corporation, United States). Student′s *t*‐test was performed for comparison among multiple groups. Statistical analysis in bioinformatics was performed using the R language (Version 4.3.0). In all analyses, *p* < 0.05 was considered statistically significant.

## 3. Results

### 3.1. SSG Inhibits Lung Cancer Proliferation Through Antitumor Mechanisms Involving TAMs

#### 3.1.1. SSG Inhibited the Growth of Lewis Lung Cancer Cells in Xenograft Mouse Model

To investigate the effects of SSG in vivo, C57 mice were subcutaneously injected with 2 × 10^6^ Lewis cells into the right armpit. After inoculation, SSGs were administered via gavage daily for 3 weeks. Doses in the mouse groups were distributed as low, medium, and high (0.52, 1.04, and 2.08 g/kg/day; *n* = 10). Cyclophosphamide (4 mg/mL) was administered via intraperitoneal injection every 4 days as a positive control (40 mg/kg; *n* = 10). Mice in the blank control group were administered via purified water gavage. The SSG‐M, SSG‐H, and CTX groups showed significant tumor suppression when compared with the blank control group. Furthermore, the growth of tumor volume of the SSG‐M, SSG‐H, and CTX groups was found to be much slower (Figure 1a,b). Compared with the model group, the SSG‐H group significantly suppressed the growth of lung cancer xenograft tumors (*p* < 0.05). There was no significant difference in the tumor weight between the SSG‐H group and the CTX group (*p* > 0.05), suggesting that the inhibitory effect of high‐dose SSG is comparable to that of 40 mg/kg CTX administered on Days 5, 9, 13, and 17 after modeling. Compared with the model group, there was no significant difference in the tumor weight of the SSG‐L group (*p* > 0.05), indicating that the application of half the dose of SSG has no significant inhibitory effect on tumor growth (Figure 1c,d).

Figure 1Comparison of transplanted tumor volume in each group of mice. (a) Volume line graph of the transplanted tumor in each group. (b) Line chart of tumor volume growth rate for each group of mice. (c) Gross morphological appearance of the transplanted tumor tissue of each group of mice. (d) Comparison of the quality of the transplanted tumor in each group of mice. Compared to the model group,  ^∗^ *p* < 0.05.

**Figure:**
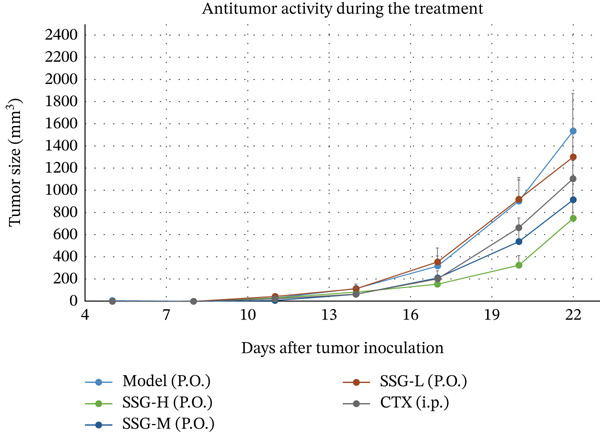
(a)

**Figure:**
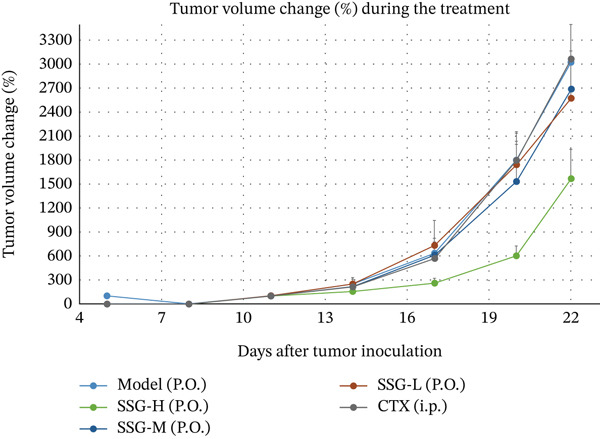
(b)

**Figure:**
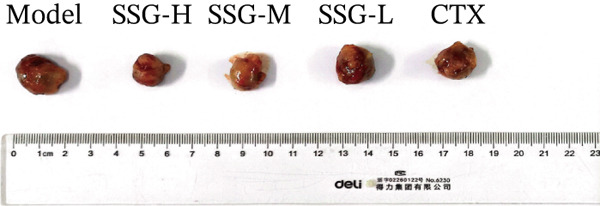
(c)

**Figure:**
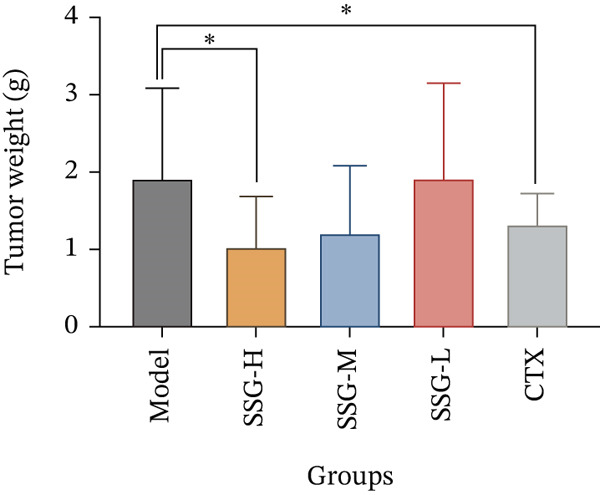
(d)

#### 3.1.2. SSG Regulated TAM Polarization In Vivo

As SSG could not inhibit lung tumor cells directly in cell proliferation assays, which was inconsistent with result demonstrated in vivo, there might be some other reasons to be explored. Tumor immunosuppressive microenvironment promotes tumor growth by inhibiting the role of immune killer cells. Measures that can reverse the tumor immunosuppressive microenvironment, such as immune checkpoint inhibitors, are the most recent antitumor research direction. Tumor‐associated macrophages constitute a substantial proportion of immune cells in the tumor microenvironment, so we hypothesized that SSG impaired the progression of tumor cells by regulating TAM phenotype and function. We detected the phenotype of TAMs in the spleen of each group via flow cytometry. After the intervention of SSG, the proportion of F4/80^+^CD206^+^ TAMs was significantly reduced, while the proportion of F4/80^+^CD206^−^ TAMs increased in SSG high‐, medium‐, and low‐dose groups compared with the blank model group (Figure 2a).

Figure 2Proportion of macrophages in the spleens of each group of mice. (a) (A) Flow cytometry graphs of F4/80^+^CD206^+^ and F4/80^+^CD206^−^ macrophages in the spleens of each mouse group. (B) Immunofluorescence method to observe the expression of CD206 in the tumor and surrounding tissues of mice in each group. (C) Comparison of the F4/80^+^CD206^+^ macrophage ratio in the spleen of mice in each group. (D) Comparison of F4/80^+^CD206^−^ macrophage ratio in the spleen of mice in each group. (E) Comparison of CD206 expression in tumor and surrounding tissues of mice in each group. (b) (A) Flow cytometry graphs of F4/80^+^CD206^−^CD86^+^ macrophages in the spleens of each mouse group. (B) Immunofluorescence method to observe the expression of CD86 in each group of mouse tumors and surrounding tissues. (C) Comparison of the proportion of F4/80^+^CD206^−^CD86^+^ macrophages in the spleen of mice in each group. (D) Comparison of CD86 expression in each group of mouse tumors and surrounding tissues. Compared to the model group,  ^∗^ *p* < 0.05,  ^∗∗^ *p* < 0.01, and  ^∗∗∗^ *p* < 0.001.

**Figure:**
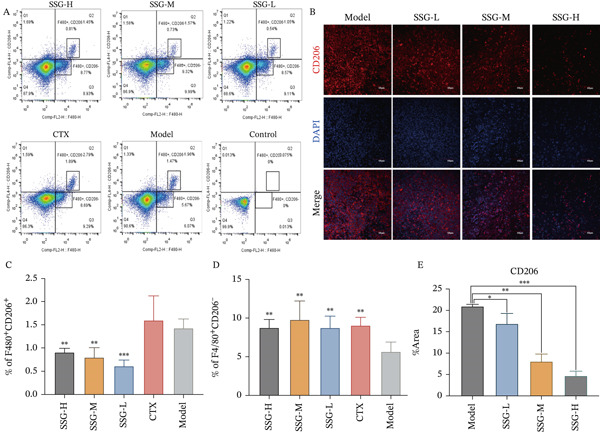
(a)

**Figure:**
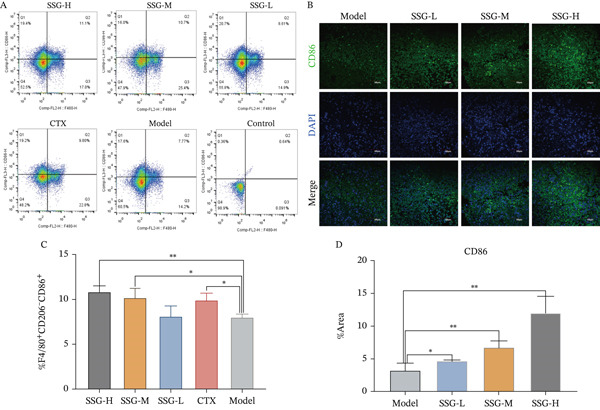
(b)

We continued to label the F4/80^+^CD86^+^ cells in the F4/80^+^CD206^+^ cell mass. The proportion of F4/80^+^CD206^−^CD86^+^ TAMs in the SSG‐treated groups was significantly higher than that in the blank model group, but the significant difference was only shown in the high‐ and medium‐dose groups. In the CTX‐treated group, the proportion of F4/80^+^CD206^+^ TAMs increased not only more significantly than in the SSG‐treated group but also than in the blank control group. The expression level of F4/80^+^ CD206^−^CD86^+^ TAMs also went high in the CTX group and was similar to that in the SSG‐M group (Figure 2b). We further investigated the M1/M2 TAM markers in mouse tumor tissues via immunofluorescence staining. It was shown that SSG could increase the expression of CD86 and downregulate CD206 expression in a dose‐dependent manner (Figure 2a,b). As a high dose of SSG was superior in tumor inhibition and M1‐like TAM regulation, SSG‐H was used in later research.

To confirm our results, we coinjected Lewis mouse cell lines with M1‐polarized macrophages and treated the model with SSG. M1‐polarized macrophage was successfully induced for later use (Figure 3a, A). Both SSG treatment and M1 macrophage coinjection could significantly inhibit tumor volume and weight, and the inhibition effects were similar. The tumor burden continually reduced in the coinjection group when treated by SSG (Figures 3a, B–D). We further investigated the TAM phenotype in coinjection and SSG‐treated models. CD86 expression increased and showed a similar proportion in M1 macrophage coinjected and SSG‐treated groups (Figure 3c, A). These results suggest that SSG can increase the proportion of M1‐type TAMs in the Lewis lung cancer mouse model and also increase the proportion of M1‐type TAMs in the lung cancer mouse model coinjected group. There was a similar expression of CD206 between control and coinjection groups, but SSG significantly reduced that in the groups of tumor‐bearing and macrophage coinjection. In terms of iNOS expression in M1‐like macrophage marker protein, IHC staining results show that the Lewis+SSG group had the highest number of cells with high iNOS expression in the transplanted tumor tissue. The IOD values of iNOS expression in IHC sections indicate that, compared with the Lewis group, the number of cells expressing iNOS in the Lewis+SSG group significantly increased, with a statistically significant difference (*p* < 0.001), suggesting that the dual parameter particles have the effect of promoting TAM polarization into M1‐like TAMs (Figure 3b, B;c, B). Taking these together, it is indicated that SSG could inhibit mouse lung ADC progression by regulating TAMs in vivo.

Figure 3Experimental diagram of SSG intervention in a mouse model of mixed transplanted tumor‐bearing M1 macrophages and Lewis lung cancer cells. (a) (A) RAW264.7 proportion of F4/80^+^CD86^+^ macrophages induced by cytokines. (B) Gross morphological appearance of the transplanted tumor tissue of each group of mice. (C) Volume graphs of transplanted tumors in each group of mice at different time points. (D) Line graph of the growth rate of transplanted tumor volume at different time points in each group of mice. (b) (A) Proportion of F4/80^+^CD86^+^ TAMs in the spleen of mice in each group. (B) IHC detection of iNOS expression in transplanted tumor tissues of mice in each group. (c) (A) Comparison of F4/80^+^CD86^+^ TAM ratio in the spleen of mice in each group (compared to the Lewis group,  ^∗^ *p* < 0.05 and  ^∗∗^ *p* < 0.01; compared to the Lewis+M1 group, ★*p* < 0.05). (B) Comparison of IOD values of iNOS expression in tumor tissues of mice in each group (compared to the Lewis group,  ^∗^ *p* < 0.01; compared to the Lewis+M1 group,  ^∗∗^ *p* < 0.001).

**Figure:**
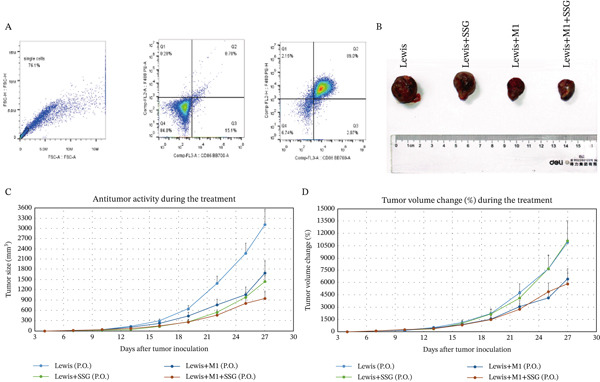
(a)

**Figure:**
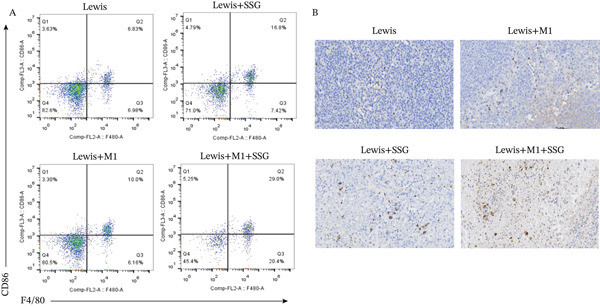
(b)

**Figure:**
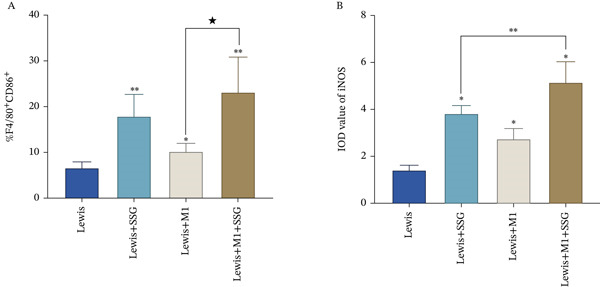
(c)

### 3.2. SSG Inhibits the Tumor‐Promoting Function of TAMs

#### 3.2.1. SSG Inhibited TAM‐Induced Protumor Factors

TAMs ensure an immunosuppressive microenvironment by inducing cytokines or factors such as Arg1, TGF‐*β*, MMP‐9, and VEGF to promote tumor progression. High levels of TGF‐*β* contribute to suboptimal antitumor immune responses, decrease T cell tumor immunity, and affect checkpoint inhibitors or cytotoxic drugs [13]. MMP‐9 and VEGF impair the extracellular stroma and vascular endothelium, thereby facilitating tumor invasion and abnormal angiogenesis [14]. To see whether SSG could affect these protumor factors mediated by TAMs, we tested their expression in tumor tissues. After treatment with SSG or coinjection with M1 macrophage, the mRNA and protein expressions of TGF‐*β*, MMP‐9, and VEGF in tumor tissues were downregulated (Figure 4a). iNOS and Arg1 are a pair of opposing biomarkers for M1/M2 macrophages, influencing tumor progression by regulating immune enzyme metabolisms [15]. The mRNA and protein expressions of iNOS were increased, while those of Arg1 were downregulated in SSG‐treated and coinjected groups when compared to the control group (Figure 4a). The results indicated that SSG inhibited lung tumor progression by impairing the expression of protumor factors mediated by TAMs.

Figure 4Effects of SSG on TAM cell functional factors, memory T cells, and Tregs in mice. (a) (A) Western blot detection bands of MIF, MMP‐9, VEGF, and TGF‐*β* in mouse tumor and peritumoral tissue. (B) Comparison of MIF protein expression. (C) Comparison of MMP‐9 protein expression. (D) Comparison of VEGF protein expression. (E) Comparison of TGF‐*β* protein expression. (F) Western blot detection bands of iNOS and ARG1 in mouse tumor and peritumoral tissue. (G) Comparison of ARG1 protein expression. (H) Comparison of iNOS protein expression. (b) (A) Flow cytometry detection charts of CD4^+^CD25^+^ T cells and CD4^+^CD25^+^Foxp3^+^ cells in the spleens of each group of mice. (B) Comparison of CD4^+^CD25^+^ T cell ratios in each group of mice. (C) Comparison of CD4^+^CD25^+^Foxp3^+^ T cell ratios in each group of mice. (c) (A) Flow cytometry detection charts of CD8^+^CD44^+^CD62^−^ memory T cells and CD8^+^CD44^+^CD62^+^ memory T cells in the spleens of each mouse group. (B) Comparison of the proportion of CD8^+^CD44^+^CD62^+^ memory T cells in each group. (C) Comparison of the proportion of CD8^+^CD44^+^CD62^−^ memory T cells in each group. Compared to the model group,  ^∗^ *p* < 0.05,  ^∗∗^ *p* < 0.01,  ^∗∗∗^ *p* < 0.001, and  ^∗∗∗∗^ *p* < 0.0001.

**Figure:**
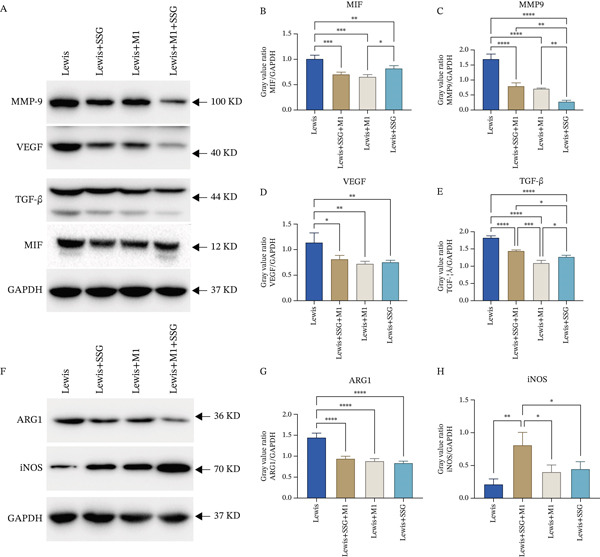
(a)

**Figure:**
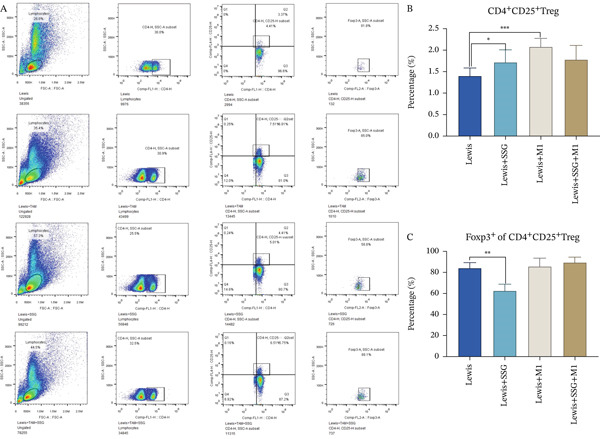
(b)

**Figure:**
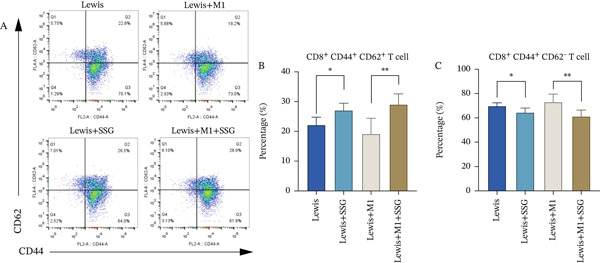
(c)

#### 3.2.2. SSG Regulated Tregs and TCMs In Vivo

TAMs had a positive correlation with increased Foxp3^+^ Treg cells and a crosstalk to CD8^+^ T cells in the tumor microenvironment [16, 17]. Foxp3^+^ regular T cells are an important immunosuppressive subpopulation. CD8^+^CD44^+^ memory T cells provide continuous tumor immune surveillance and killing ability, which is the hotspot in recent years. We tested Foxp3 expression in CD4^+^CD25^+^ Treg cells and CD8^+^CD44^+^CD62^+/−^ memory T cells in the tumor environment. Flow cytometry analysis revealed that the proportion of CD4^+^CD25^+^ Treg cells in the control group ranked the lowest among all four groups. After being treated with SSG, the proportion of CD4^+^CD25^+^ Treg cells increased compared with the control group, and that in the M1 coinjected group presented a more noticeable upward tendency (Figure 4b). Concerning changes made by different interventions on the ratio of Foxp3^+^ Treg cells, only the ratio in the SSG‐treated group decreased, while the other two groups showed no obvious changes (Figure 4b). The results illustrated that SSG can reduce the ratio of CD4^+^CD25^+^Foxp3^+^ Treg cells in the tumor microenvironment.

As CD8^+^CD44^+^CD62^−^ memory T cells are effector memory T cells (TEMs) and CD8^+^CD44^+^CD62^+^ memory T cells are central memory T cells (TCMs), the ratio of CD62^−^ memory T cells in the control group was larger than that of CD62^+^ memory T cells (Figure 4c). Compared with the control group, SSG treatment improved the ratio of TCMs and downregulated that of TEMs. On the other hand, although the ratio of TCMs decreased and that of TEMs increased in the M1 coinjected group, no significant difference was found. When comparing the group treated with both SSG and M1 macrophage with the M1 coinjected group, the ratio of TCMs showed a remarkable increase, while the ratio of TEMs exhibited an opposite change pattern. Therefore, SSG was demonstrated to upregulate the ratio of CD8^+^CD44^+^CD62^+^ memory T cells and downregulate that of CD8^+^CD44^+^CD62^−^ memory T cells.

### 3.3. SSG Suppresses MIF Expression in Lewis Cancer Cells by Promoting the Expression Level of miR‐34a in Tumor‐Derived Exosomes

#### 3.3.1. SSG Inhibited MIF Expression in Lewis Cells

As MIF expression had an effect on the phenotype of TAMs through regulating the tumor‐derived exosomes, we investigated whether SSG could influence MIF expression in Lewis cells. Since the Chinese medicine compound works after being absorbed and metabolized into the blood, we prepared an SSG drug–containing serum. Firstly, MIF mRNA overexpression and MIF mRNA knockdown cell lines (MIF^+/+^ Lewis cell and shMIF Lewis cell) were established via plasmid transfection. Subsequently, wild‐type, MIF^+/+^, and shMIF Lewis cell lines were each treated with an SSG drug–containing serum. SSG was observed to inhibit both mRNA and protein expression of MIF in these Lewis cell lines (Figure 5a). The results above suggest that SSG had an inhibitory effect on MIF of Lewis cells in vitro.

Figure 5SSG suppresses MIF expression in Lewis cancer cells by promoting the expression level of miR‐34a in tumor‐derived exosomes. (a) A Western blot detection of MIF protein expression in each group of cells. B, D Comparison of MIF protein expression in each group of cells. C, E Comparison of the relative expression levels of MIF RNA in each group of cells using the qPCR method. (b) A Gross morphological appearance of the transplanted tumor tissue of each group of mice. B Condition of tumor weight in each group of mice. C Volume line graph of the transplanted tumor in each group.

**Figure:**
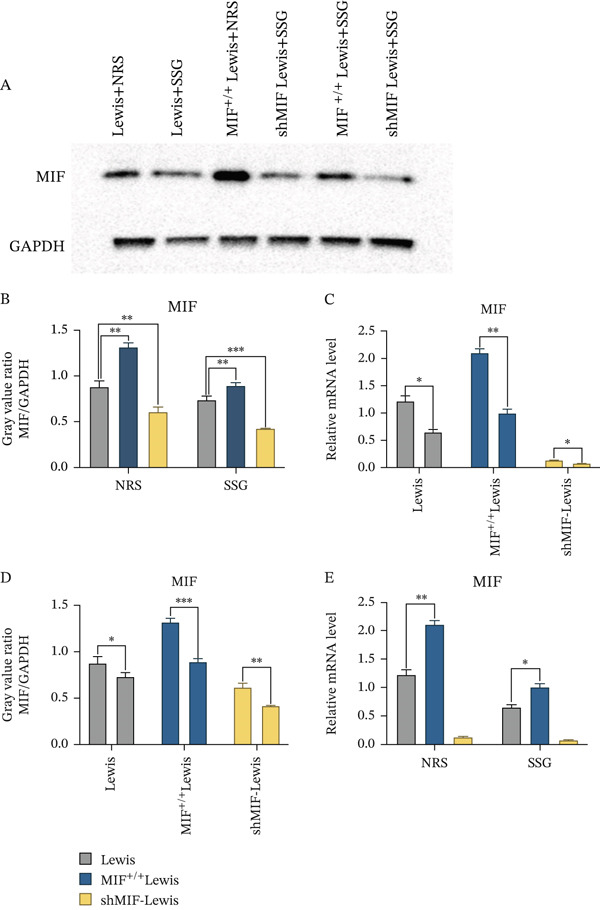
(a)

**Figure:**
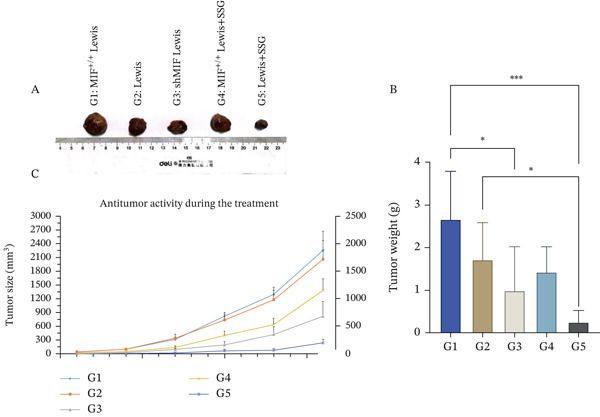
(b)

In order to further clarify the mechanism of SSG, we injected 2 × 10^6^ MIF^+/+^ Lewis cells, shMIF Lewis cells, and Lewis cells into the right armpit of mice according to groups, respectively. Combined with the research scheme, SSG was used to deal with the corresponding model. Studies have shown that injection of MIF^+/+^ Lewis cells promotes tumor growth in a mouse lung cancer model compared to normal Lewis cells, while SSG treatment inhibits the effects of MIF overexpression and reduces tumor volume and tumor weight in mice (Figure 5b).

#### 3.3.2. SSG Promoted the Expression of Biomarkers on Exosomes and Increased the Expression Level of miR‐34a in MIF^+/+^ Lewis Cells

As exosomes containing microRNAs could regulate macrophage phenotype and be affected by MIF [18], microRNA‐34a, which can also be inhibited by MIF [19], is considered an important inhibiting factor for tumorigenesis and M2 macrophage polarization [20]. Therefore, we detected the expression level of miR‐34a in tumor‐derived exosomes. To explore the effect of SSG treatment on tumor‐derived exosomes and the expression level of miR‐34a, we extracted exosomes from the culture medium of wild‐type, MIF^+/+^, shMIF, and SSG‐treated MIF^+/+^ Lewis cell lines. Firstly, we thoroughly observed and measured the structure of the extracted material to ensure proper collection of exosomes (Figure 6a). The Brownian motion of exosomes was also recorded to analyze their mobility. Secondly, the biomarkers of exosomes CD9, CD63, and TSG101 were tested. Lastly, the expression level of miR‐34a was detected. The Brownian motion of exosomes was found to be more active in the SSG‐treated group. The biomarkers showed a higher expression in the SSG‐treated group but no significant differences among the other groups (Figures 6b, 6c, and 6d). The level of miR‐34a in tumor‐derived exosomes ranked the lowest in the MIF^+/+^ Lewis cell group. The expression level of miR‐34a was elevated in both the shMIF and SSG‐treated groups. The results indicated that SSG regulated MIF expression by increasing the level of miR‐34a.

Figure 6The drug‐containing serum of SSG affects Lewis‐origin exosomes and miR‐34a in exosomes. (a) Electron microscopy images of exosomes from each group and the exosome Brownian motion recording graph. (b) Banding diagram of each group of exosome marker proteins CD9, CD63, and TSG101. (c) Comparison of miR‐34a expression in each group of exosomes. (d) Exocrine particle size detection chart of each group.

**Figure:**
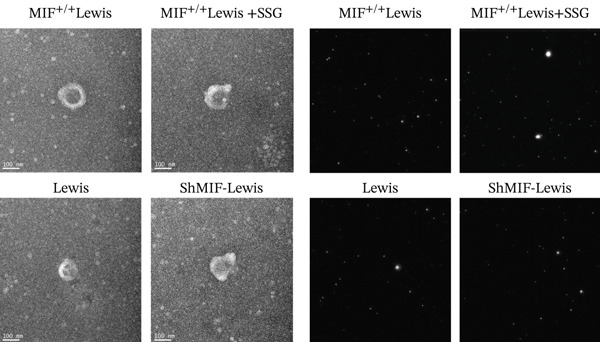
(a)

**Figure:**
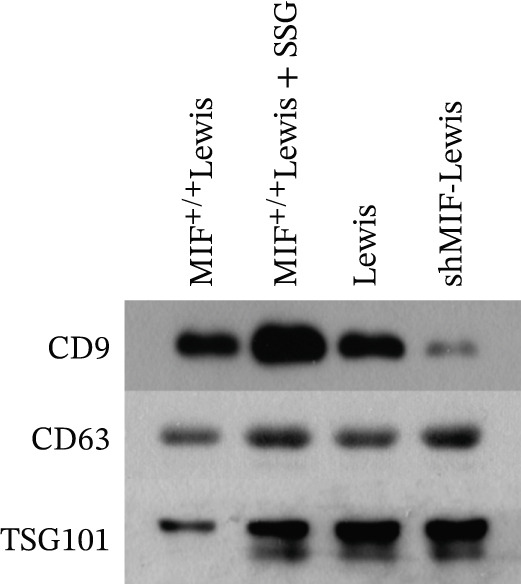
(b)

**Figure:**
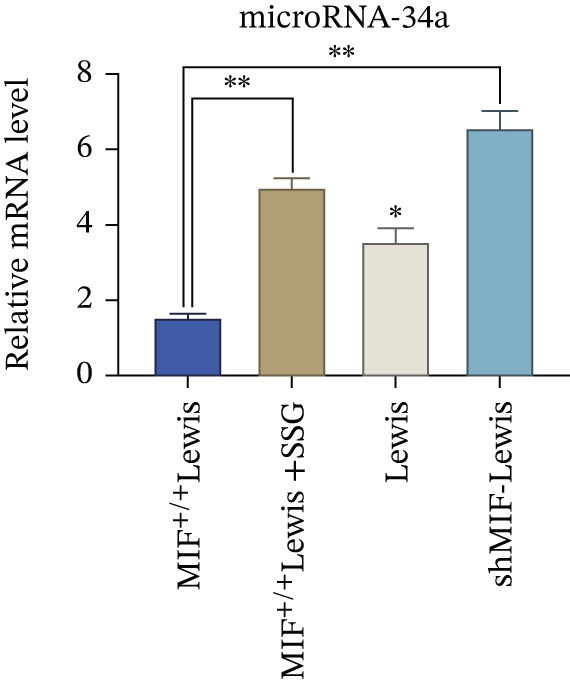
(c)

**Figure:**
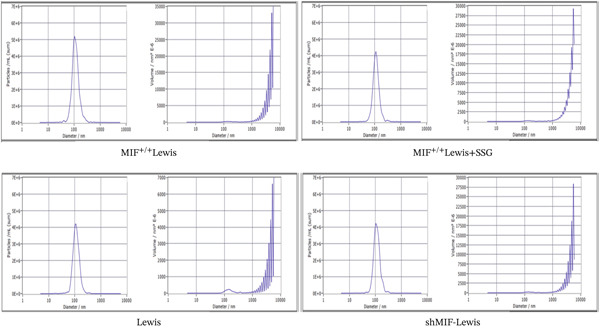
(d)

### 3.4. SSG Can Inhibit the Polarization Change of TAMs Caused by the Overexpression of MIF, and Its Mechanism Is Related to the MIF‐miR‐34a‐KLF4 Pathway

#### 3.4.1. The Expression of MIF Influenced the Phenotype of TAMs by Regulating Tumor‐Derived Exosomes, and SSG Was Able to Suppress This Effect

Many factors affect the differentiation of macrophages. Exosomes play a crucial role in intercellular communication and mediating interactions between tumor cells and TAMs [21]. To investigate the effect of exosomes derived from Lewis cells on TAM phenotype, exosomes from the Lewis cell line culture medium were extracted and cocultured with the mouse monocyte–macrophage RAW264.7 cell line. Before coculturing, LPS and IFN‐*γ* were used to induce M1 macrophages, serving as one control group, while M2 macrophages in another control group were induced using IL‐4 and IL‐13 (Figure 7a). The biomarkers of the different phenotypes of TAMs were also detected to verify the successful induction of both M1 and M2 macrophages for later use. The results showed that tumor‐derived exosomes were phagocytosed by RAW264.7 cells, resulting in the downregulation of CD86 expression in these cells (Figure 7b). As MIF could remodel TAMs, we further detected how exosomes derived from tumor cells, which were mediated by MIF, could affect TAMs. Exosomes derived from tumor cells were extracted from the culture supernatant of these cell lines and incubated with RAW264.7 cells. RAW264.7 cells incubated with exosomes derived from MIF^+/+^ Lewis cells exhibited a less M1 and more M2 phenotype compared to those incubated with exosomes from Lewis wild‐type cells (Figure 7c). On the other hand, when incubated with exosomes derived from shMIF Lewis cells, RAW264.7 cells showed more M1 and less M2 phenotype. These results indicated that MIF expressed by Lewis cells could induce tumor‐derived exosomes that further lead TAMs to the M2 phenotype. In order to test how SSG could make a difference to the effect proved above, we further added one group where exosomes were derived from MIF^+/+^ Lewis cells treated by SSG. It was demonstrated that SSG could inhibit the evolution of TAMs toward an M2 phenotype while facilitating the M1 polarization of TAMs (Figure 7d). The effect of SSG on the polarization of TAMs was similar to that observed in the shMIF Lewis cells group. The results indicated that SSG could suppress the alterations in TAM polarization caused by MIF overexpression.

Figure 7Effects of SSG‐containing serum on macrophage phenotype and KLF4 of MIF^+/+^ Lewis cell–derived exosomes. (a) A Megafluorescence image of Lewis cell–derived exosomes internalized by macrophages. B The control group was induced by cytokines after F4/80^+^CD206^+^ and F4/80^+^CD86^+^ (flow cytometry graph). (b) A iNOS and ARG1 protein bands. B F4/80^+^CD86^+^ cell flow histogram after 24 h of coculture. (c) F4/80^+^CD206^+^ cell scale chart after 24 h of total culture. (d) Comparison of F4/80^+^CD86^+^ macrophage ratio after coculture. (e) KLF4 RNA relative expression level. (f) TNF‐*α* RNA relative expression level. (g) IL‐10 RNA relative expression level. Compared with the MIF^+/+^ Lewis group,  ^∗^ *p* < 0.05,  ^∗∗^ *p* < 0.01, and  ^∗∗∗^ *p* < 0.001.

**Figure:**
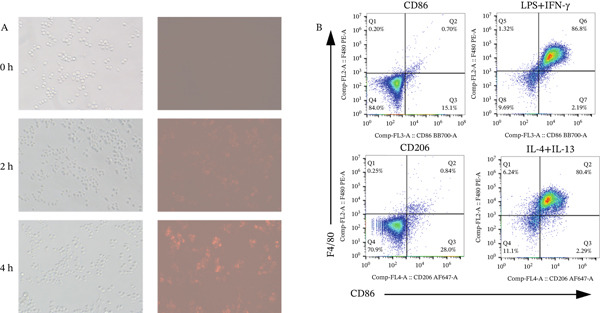
(a)

**Figure:**
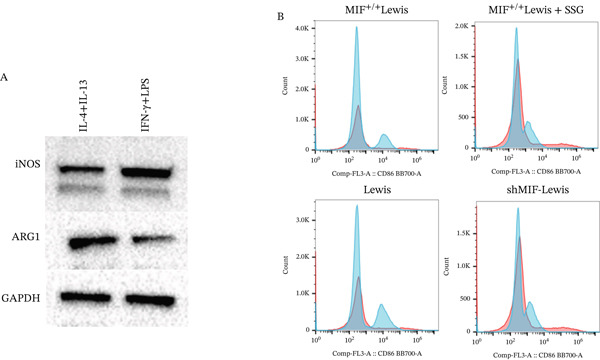
(b)

**Figure:**
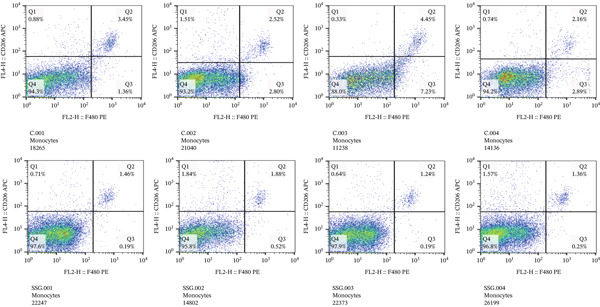
(c)

**Figure:**
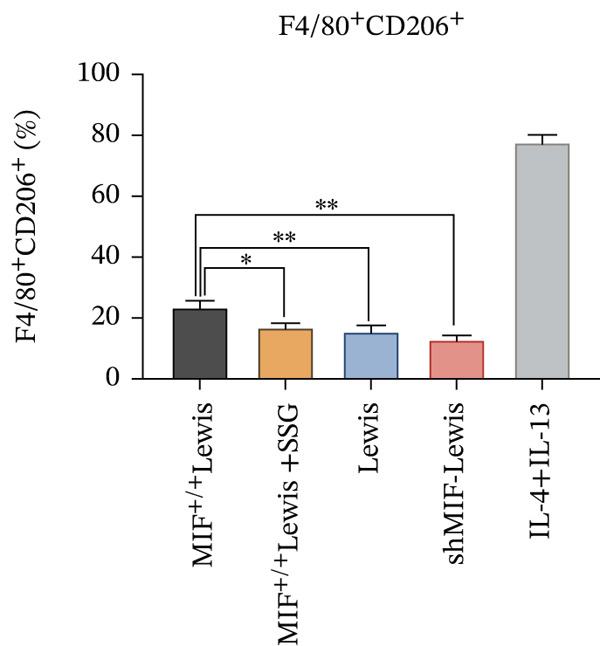
(d)

**Figure:**
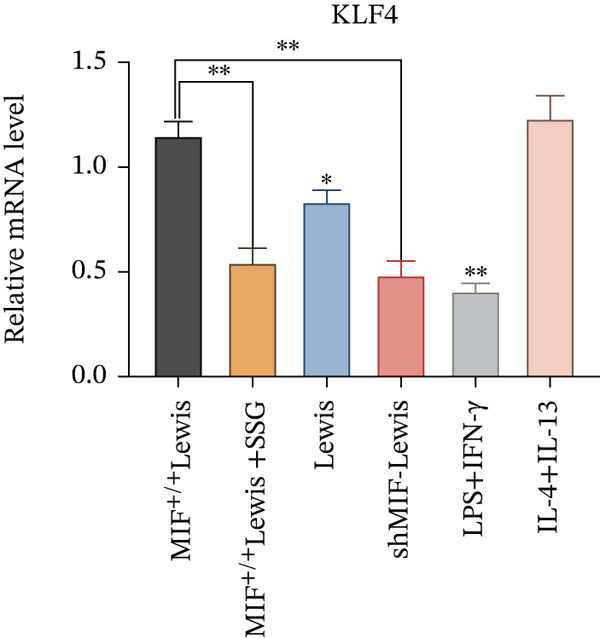
(e)

**Figure:**
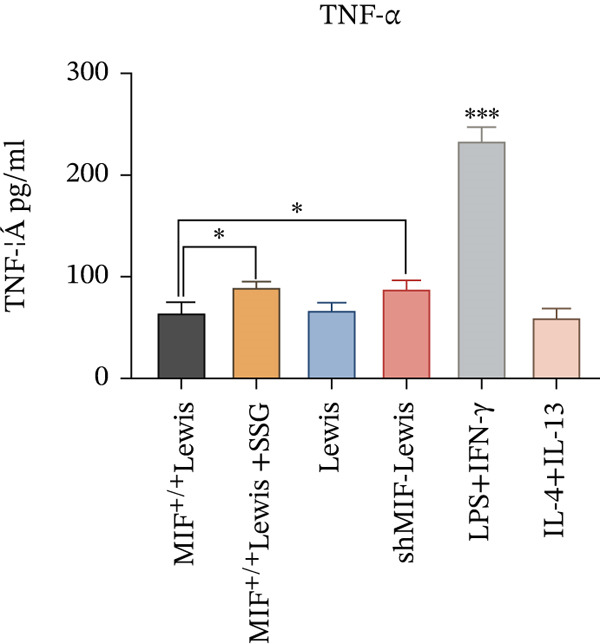
(f)

**Figure:**
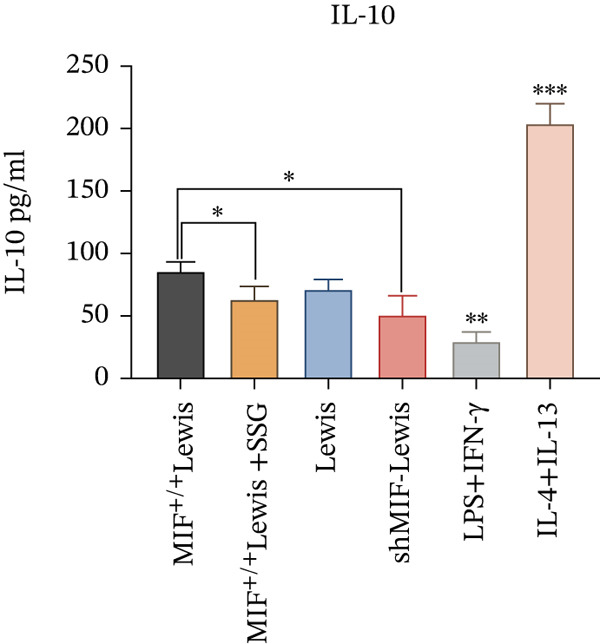
(g)

#### 3.4.2. SSG Remodeled Phenotype of TAMs via Inhibiting the KLF4 Pathway

To further investigate the mechanism of SSG on remodeling TAMs, we focused on the MIF‐related signal pathways. Though MIF exosomes can be produced by some tumor cells [22], little MIF was detected in exosomes secreted by Lewis cells or A549 cells. As research showed that MiR‐34a induced M1 macrophage phenotype polarization via targeting KLF4 3 ^′^‐untranslated region and decreasing KLF4 expression [23], we tested KLF4 expression in macrophages incubated with exosomes. After incubation with exosomes derived from tumor cells, the mRNA of KLF4 expression in TAMs significantly decreased in the SSG‐treated Lewis cell group (Figure 7e). The concentration of TNF‐*α* and IL‐10 was detected, as TNF‐*α* is one of the cytokines specifically secreted by M1 macrophages and IL‐10 by M2 macrophages. Compared with the MIF^+/+^ Lewis cell group, the concentration of TNF‐*α* rose significantly in the SSG‐treated group; a similar promoting effect was also observed in the shMIF Lewis cell group (Figure 7f). With regard to the concentration of IL‐10, SSG treatment could inhibit the promoting effect produced by MIF^+/+^ Lewis cells on IL‐10 expression (Figure 7g). The results indicated that the mechanism of SSG regulating TAM polarization is closely related to the MIF‐miR‐34a‐KLF4 pathway.

### 3.5. Identification of the Ingredient Characterization of SSG

**Figure 8:**
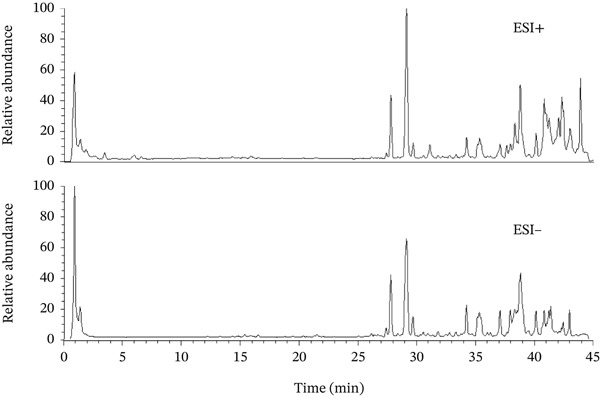
Total ion chromatograph (TIC) of the chemical extract of SSG.

A total of 125 constituents were identified from Shuangshen granule based on the UHPLC‐Q‐Orbitrap HRMS technique. Mainly, these include saponins (*Panax* saponin Rk3, *Panax notoginseng* saponin J, American ginseng saponin L1, *Cynanchum atratum*, *Panax* saponin Rg5, etc.), flavonoids (quercetin‐3‐O‐*β*‐D‐galactopyranosyl [2⟶1] glucoside, ginsenoside, saponin, kaempferol‐3‐O‐sambubioside, isorhamnetin, etc.), nucleotides (cordycepin, xanthine, uridine, cytidine, cytosine, etc.), amino acids (*Panax notoginseng* saponin, L‐arginine, *Panax notoginseng* saponin, L‐isoleucine, phenylalanine, etc.), and other components. One hundred and twenty‐five chromatographic peaks are shown in the total ion chromatograph (TIC) (Figure 8).

### 3.6. Bioinformatics Prediction

#### 3.6.1. The Difference Between Mouse Exocrine and LUAD miRNA Intersection

For the miRNA detection data of 527 cases of LUAD, the R package “limma” was used to perform differential comparison analysis between cancer and normal, to obtain differentially expressed genes. The differential screening threshold was adj.*p*.val < 0.05 and (|log FC|) > 1, resulting in 179 differentially expressed miRNAs, with 134 upregulated and 45 downregulated. Heat maps and volcano plots were used to visualize the differentially expressed miRNAs separately (Figure 9a, A and B). Based on the differential miRNA expression profile of mouse exosomes, differential miRNAs with log2.Fold_change. were screened. In the case of |log2.Fold_change.| > 4, among the 157 differential miRNAs, 132 were upregulated, and 25 were downregulated. Based on the filtered LUAD differential miRNA and mouse exosome differential miRNA (|log2.Fold_change.| > 4), an intersection was obtained, resulting in 17 intersecting miRNAs, which were then displayed in a Venn diagram (Figure 9a, C). Through this part of bioinformatics analysis, it was predicted that miR‐34a in this experiment is miR‐34a‐5p (Table 3).

Figure 9Bioinformatics analysis diagram. (a) A LUAD differential miRNA, blue indicates lower expression, red indicates higher expression, and gray indicates no difference. B Differential miRNA heat map. C Mouse exosome and LUAD differential miRNA intersection Venn diagram. (b) A M0‐like macrophage content. B M1‐like macrophage content. C M2‐like macrophage content. D Differences in macrophage infiltration groups. E Analysis of the correlation between miR34a‐5p and regulatory genes with immune cells.

**Figure:**
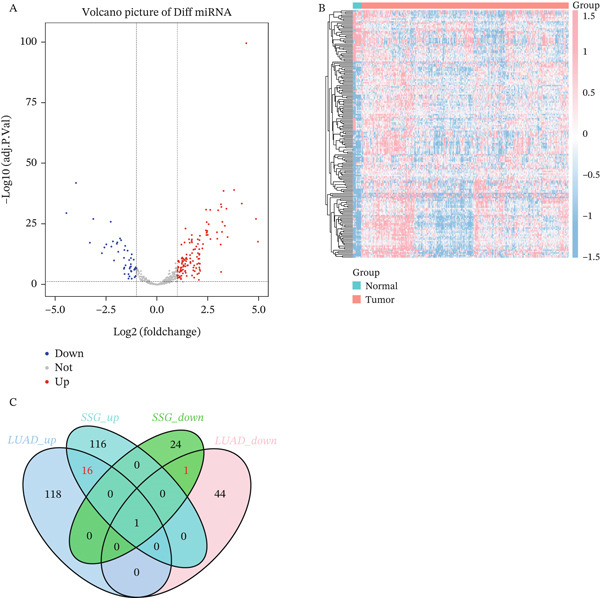
(a)

**Figure:**
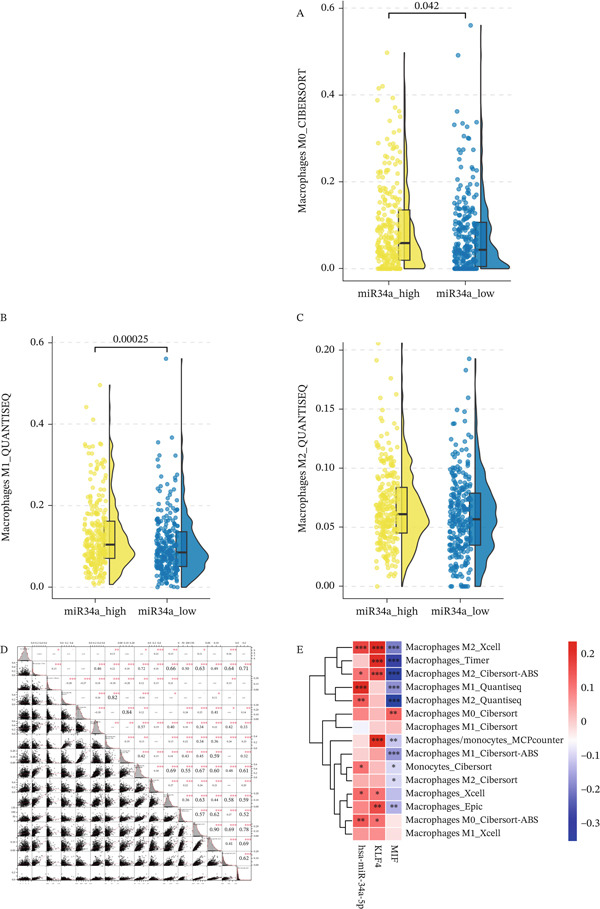
(b)

**Table 3:** Different intersecting miRNAs.

miRNA	Style	*l* *o* *g* *F* *C*	sRNA	SSG_H
hsa‐miR‐148b‐5p	Up	1.047136594	mmu‐miR‐148b‐5p	11.56001544
hsa‐miR‐17‐3p	Up	1.224120311	mmu‐miR‐17‐3p	11.56001544
hsa‐miR‐18a‐5p	Up	1.495657536	mmu‐miR‐18a‐5p	52.02006948
hsa‐miR‐193a‐5p	Down	−1.272407557	mmu‐miR‐193a‐5p	0
hsa‐miR‐193b‐3p	Up	1.006600874	mmu‐miR‐193b‐3p	28.9000386
hsa‐miR‐194‐5p	Up	1.722881784	mmu‐miR‐194‐5p	109.8201467
hsa‐miR‐19a‐3p	Up	2.626417252	mmu‐miR‐19a‐3p	75.14010037
hsa‐miR‐19b‐3p	Up	2.464481499	mmu‐miR‐19b‐3p	1173.341567
hsa‐miR‐20b‐5p	Up	1.460563139	mmu‐miR‐20b‐5p	11.56001544
hsa‐miR‐29a‐5p	Up	2.109370939	mmu‐miR‐29a‐5p	40.46005404
hsa‐miR‐32‐5p	Up	1.360122672	mmu‐miR‐32‐5p	132.9401776
hsa‐miR‐345‐5p	Up	1.275088195	mmu‐miR‐345‐5p	34.68004632
hsa‐miR‐34a‐5p	Up	1.418686177	mmu‐miR‐34a‐5p	57.8000772
hsa‐miR‐34b‐5p	Up	1.181104834	mmu‐miR‐34b‐5p	11.56001544
hsa‐miR‐409‐5p	Up	1.413013423	mmu‐miR‐409‐5p	11.56001544
hsa‐miR‐582‐3p	Up	1.783437536	mmu‐miR‐582‐3p	63.58008492
hsa‐miR‐9‐5p	Up	4.956448082	mmu‐miR‐9‐5p	7005.369357

#### 3.6.2. Analysis of the Correlation Between miR34a‐5p and mRNA in Immune Cells

Combining the results of the LUAD sample immune infiltration analysis in TIMER, the data shows that there are statistically significant differences in the expression of miR34a‐5p between the high‐expression and low‐expression groups in monocytes, M0 macrophages, M1 macrophages, and M2 macrophages. Both M1 and M2 macrophages show higher contents in the high‐expression group, but the statistical result analysis shows that the proportion of M1 macrophages is higher in the miR34a‐5p high‐expression group, and the proportion of M1 macrophages is higher in the miR34a‐5p low‐expression group (Figure 9b, A–C). In terms of immune infiltration analysis, miR34a‐5p has the highest Pearson correlation coefficient with M1 macrophages, with a value of 0.21, suggesting the potential ability of miR34a‐5p to influence the related functions of M1 macrophages, which is consistent with our experimental results above (Figure 9b, D). In terms of genetics, we studied the MIF and KLF4 genes involved in this experiment and found that both genes are related to M1 and M2 macrophages. The results indicate that miR34a‐5p can affect the polarization of macrophages to M1 macrophages, and the functions of the MIF and KLF4 genes involved in this process have also been validated twice in bioinformatics (Figure 9b, E).

## 4. Discussion

**Figure 10:**
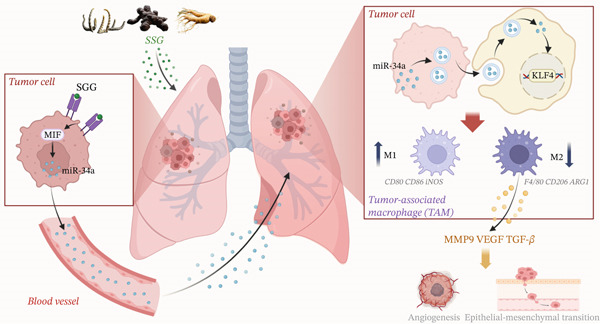
A schematic outlining the possible mechanisms by which SSG suppresses lung cancer by regulating MIF‐miR‐34a‐KLF4 signaling pathways. The compound Chinese medicine SSG, composed of *Cordyceps sinensis*, American ginseng, and *Panax notoginseng*, acts on the MIF gene locus of tumor cells to regulate the synthesis of miR‐34a. miR‐34a is then transported to TAMs by exosomes. After TAMs engulf the exosomes, the miR‐34a within the exosomes further regulates the KLF4 gene, leading to an increase in M1‐type macrophages and a decrease in M2‐type macrophages, thereby exerting an anti–lung cancer therapeutic effect.

The early symptoms of lung cancer are not obvious, and it has the characteristics of high morbidity and high mortality [24]. The clinical treatment of lung cancer has always been a difficult problem in the medical field. Although new therapeutic targets and strategies have emerged in recent years, it is highly likely that chemoresistance to conventional anticancer therapies has severely affected the survival outcome of a large proportion of patients. Therefore, there is an urgent need to explore new treatment strategies to improve the survival rate and quality of life of patients with lung cancer. Based on the theory of traditional Chinese medicine, SSG has a good effect on the prevention and treatment of lung cancer by supplementing qi, nourishing yin, and promoting blood circulation. This study proves that SSG has a regulatory effect on TAMs by regulating the tumor cell MIF‐MiR‐34a exosome pathway, controlling the polarization and function of TAMs (Figure 10).

As an important role in the coordination of immune defense and the antitumor immune mechanism, in recent years, TAMs have gradually attracted the attention of researchers [25, 26]. TAMs are derived from monocytes in bone marrow and the blood circulation, undergoing the modification of tumor cells and the tumor microenvironment. They are recognized as immune cells with plasticity, since TAMs can be activated by microenvironmental signals, exhibiting diverse functions and phenotypes in response to different activation signals [27–29]. According to different gene profiles activated by different signals, TAMs can be divided into M1 and M2 types. M1 macrophages, which express CD80 or CD86 in mice, can be induced by LPS or Helper T Cell Type 1 cytokines such as IFN‐*γ*, contributing to inflammatory response, immunity activation, and antitumor process [30]. On the other hand, compared with M1 macrophages, M2‐like TAMs are demonstrated in response to Th2 cytokines like IL‐5, IL‐4, and IL‐10 and exhibit opposite properties by functioning as a suppressive factor in inflammatory response and a promoting factor in tumor progression [31, 32].

M1‐like TAMs, whose main characteristics include the expression of iNOS, CD80, and CD86 markers, can produce IFN‐*γ*, TNF‐*α*, and other cytokines to mediate inflammation and inhibit the growth of tumor cells [33]. Conversely, mainly characterized by the expression of AGR1 and CD206 marker, M2‐like TAMs promote angiogenesis and epithelial–mesenchymal transformation through the release of MMP‐9, VEGF, and TGF‐*β* [34]. In this study, western blot was used to detect the expression of iNOS, ARG1, MMP‐9, TGF‐*β*, MIF, and VEGF. The analysis results demonstrated that with the rise in the proportion of M1‐like TAMs, the expression of iNOS also increased significantly, while the expression of MIF, VEGF, and ARG1 decreased. Different from other cytokines, MIF is primarily expressed in lung cancer cells and macrophages, having an effect on regulating the polarization of TAMs. The results indicated an elevation in MIF expression in the tumor tissue of mice, but after the intervention of SSG, MIF expression decreased. Since both tumor cells and macrophages express MIF, further exploration is needed to determine whether SSG regulates TAM polarization through MIF.

Memory T cells are a specialized subset of T cells within the tumor immune microenvironment that play a critical role in antitumor immune responses. In mice, TCMs are characterized by the expression of CD44^+^CD62L^+^, whereas TEMs exhibit a CD44^+^CD62L^−^ phenotype. Analysis of memory T cells revealed that the proportion of CD8^+^ TCM in the spleen significantly increased following SSG intervention, while CD8^+^ TEM showed an opposite trend. Consistent results were observed in the M1‐like TAMs and Lewis cell cotransplantation tumor model: SSG intervention increased the proportion of CD8^+^ TCM, further confirming that SSG can enhance CD8^+^ TCM levels. Overexpression of M1‐like TAMs was found to elevate CD8^+^ TEM proportions, but SSG intervention suppressed this M1‐like TAM‐mediated effect, reducing CD8^+^ TEM levels in the M1‐like TAMs/Lewis lung cancer cotransplantation model. The impact of SSG on CD8^+^ TEM proportions positively correlated with its tumor growth inhibitory effects and M1‐like TAM levels, suggesting that SSG‐induced alterations in memory T cell subsets may be linked to its modulation of TAM polarization and function. Although TEM can release tumor‐killing IFN‐*γ*, their rapid terminal differentiation results in poor survival and limited antitumor efficacy in animal studies. In contrast, TCM demonstrates superior tumor‐killing activity. Thus, increased TCM proportions coupled with decreased TEM levels enhance tumor cell elimination and inhibit tumor growth. In this study, through the analysis of EMT factors, angiogenesis factors, immunosuppressive factors, Treg cells, and memory T cells in the microenvironment, the regulatory effect of SSG on the tumor microenvironment was preliminarily verified, and this effect was related to the proportion of M1‐like TAMs and the inhibitory effect on the tumor.

To verify whether SSG regulates TAM polarization through MIF, we constructed Lewis cell lines with MIF overexpression (MIF^+/+^) and MIF interference (shMIF). This mechanism was verified in vitro with stably transfected cell lines, showing that the metabolized drug components of SSG can downregulate MIF expression in lung cancer cells. In vivo studies, we found that overexpression of MIF significantly increased tumor size and weight, and SSG inhibited this process. Simultaneously, the pharmacological components of these herbs also influence exosome release and small RNA expression in exosomes, as MIF regulates the expression of miR‐34a in cells [19]. When the MIF gene is overexpressed in tumor cells, the expression of miR‐34a in tumor‐derived exosomes is decreased. After macrophages endocytose these exosomes, the expression of intracellular KLF4 is increased due to the loss of miR‐34a inhibition. In the meantime, the expressions of F4/80 and CD206 in macrophages were increased, while the expressions of CD86 and CD80 were downregulated, which was verified by the macrophage endocytosis exosome assay. Based on the experimental results, we concluded that MIF of tumor cells may influence the polarization of macrophages through the miR‐34a/KLF4 pathway after macrophages endocytose exosomes. KLF4 is an important gene involved in macrophage polarization. Studies have demonstrated that macrophages with KLF4 gene deletion exhibit increased M1 marker gene expression [35]. The phenotype of TAMs can be repolarized in response to cytokines in the tumor microenvironment, where they constantly adapt to changing signals [36]. KLF4 can suppress the inflammatory response in the tumor microenvironment by regulating the polarization of TAMs [37]. Therefore, decreased KLF4 expression can promote the polarization of TAMs to M1.

After the intervention of SSG, the expression of miR‐34a in tumor‐derived exosomes was increased. Subsequently, the expression of KLF4 was decreased, and the expression of F4/80 and CD86 markers was upregulated in macrophages after exosomes endocytosis. Therefore, we concluded that Lewis lung cancer cells may promote the M1‐type polarization of TAMs through exosomes that connected the MIF‐miR‐34a signal to the KLF4 target of macrophages. Previous experimental studies have shown that the KLF4 signaling pathway downstream of miR‐34a is involved in the polarization of macrophages and affects the inflammatory response [20]. Macrophages are cells that play a role in both innate immunity and adaptive immunity, serving as a bridge connecting the two kinds of immunity by influencing both the inflammatory microenvironment and the immune microenvironment in cancer diseases. Through in vitro and in vivo experiments, we explored the mechanism of SSG on the polarization of TAMs induced by Lewis‐derived exosomes, providing a preliminary basis for subsequent studies on the regulation of the tumor microenvironment by SSG.

Based on UHPLC‐Q‐Orbitrap HRMS technology, we identified 125 chemical components of SSG, including cordycepin, 5 ^′^‐deoxy‐8,5 ^′^‐cyclic adenosine monophosphate, and further clarified the material basis of SSG inhibiting lung cancer. For example, cordycepin, the representative active compound in SSG, has anti‐inflammatory, antiviral, and antitumor effects. It has been proven that it can control the progression of tumors such as lung cancer and bladder cancer by inducing apoptosis and inhibiting cell proliferation and migration [38, 39]. Liao et al.′s study showed that cordycepin can reverse cisplatin resistance in non‐small‐cell lung cancer by activating AMPK and inhibiting the AKT signaling pathway [40].

Although this study comprehensively explores the targeted therapeutic mechanism of SSG regulating TAM polarization through lung cancer cell–derived exosomes, there are still some problems to be solved in our experiments. In this study, although the chemical constituents of SSG were identified by UHPLC‐Q‐Orbitrap HRMS technology, the relevant experimental studies on the specific active ingredients were not carried out. In the future, we will carry out relevant experimental studies around the active ingredients of SSG to improve the level of experimental evidence. With the continuous deepening of modern medicine′s research on the pathogenesis of tumors, although new treatment methods and drugs have made certain progress, the high recurrence rate, metastasis risk, drug resistance, and toxic and side effects during the treatment process are still bottlenecks that need to be broken through in the medical field. Traditional Chinese medicine, with its unique theoretical system and rich practical experience, provides new ideas and methods for tumor treatment, and its future development prospects are promising. By fully analyzing the synergistic mechanism of multicomponents, multitargets, and multipathways in traditional Chinese medicine compounds, it is clarified how the active components of traditional Chinese medicine affect the proliferation, apoptosis, invasion, and metastasis of tumor cells at the levels of cell signaling pathways and gene expression regulation. With the in‐depth application of multiomics technologies such as single‐cell genomics, metabolomics, and proteomics, it is expected to further explore the potential targets and molecular networks of traditional Chinese medicine in tumor intervention, providing theoretical support for new drug development and precision treatment. In conclusion, bioinformatics‐based approaches combined with in vivo and in vitro experiments confirm that SSG can regulate TAM polarization and function by modulating MIF‐miR‐34a‐KLF4 pathway–related signals.

**Nomenclature**
TIME: tumor immunosuppressive microenvironment
TAMs: tumor‐associated macrophages
SSG: Shuangshen granule
NSCLC: non‐small‐cell lung cancer
BMCs: bone marrow cells
MDSCs: myeloid‐derived suppressor cells
SSG‐H: Shuangshen granule‐high
SSG‐M: Shuangshen granule‐median
SSG‐L: Shuangshen granule‐low
LUAD: lung adenocarcinoma
MIF: macrophage migration inhibitory factor
KLF: Krüppel‐like factor
miR: microRNA
Tregs: regulatory T cells
BSA: bovine serum albumin
DAB: diaminobenzidine
TIC: total ion chromatograph
TEMs: effector memory T cells
TIER: tumor immune estimation resource
CD206: Mannose Receptor C Type 1
CD86: leukocyte antigen 86
F4/80: EGF‐Like Module‐Containing Mucin‐Like Hormone Receptor–Like 1
ARG1: Arginase‐1
iNOS: inducible nitric oxide synthase
VEGF: vascular endothelial growth factor
TGF: transforming growth factor
IL: interleukin
TME: tumor microenvironment
Foxp3: forkhead box protein
LPS: lipopolysaccharide
CTX: cyclophosphamide
GAPDH: glyceraldehyde‐3‐phosphate dehydrogenase

## Author Contributions

Qiujun Guo, Yefan Zhang, and Bo Pang conceived and designed the study; Zhongning He, Yongxia Hou, and Baojin Hua participated in extracting data; Yuwei Zhao, Qi Huang, and Zhan Shi participated in the development and revisions of the manuscript; Runzhi Qi and Yi Li wrote the paper. All authors contributed to the writing of the final manuscript. Runzhi Qi, Qi Huang, Yi Li, and Zhongning He have contributed equally to this work.

## Funding

Funding was supported by the Beijing Natural Science Foundation (No. 7232310), the National Natural Science Foundation of China (No. 82205226), the High Level Chinese Medical Hospital Promotion Project (Nos. HLCMHPP2023085 and HLCMHPP202308506), the China Academy of Chinese Medical Sciences Outstanding Young Science, Technology Talent Training Special Project (No. ZZ15‐YQ‐026), the Chinese Association of Chinese Medicine (CACM) Youth Talent Support Project (No. CACM‐2023‐QNRC2‐B01), and the Noncommunicable Chronic Diseases‐National Science and Technology Major Project (No. 2023ZD0502500).

## Disclosure

All authors read and approved the final manuscript.

## Conflicts of Interest

The authors declare no conflicts of interest.

## Data Availability

Data supporting the findings of this study are available from the corresponding authors if reasonably requested.
